# The Influence of Planting Different Green Manures in Apple Orchards on Soil Fertilizer and Apple Quality in the Loess Plateau

**DOI:** 10.3390/plants15193073

**Published:** 2026-10-08

**Authors:** Zhiyuan Bian, Yongjuan Cheng, Yang Lu, Wenfang Li, Xin Li, Yingjun Hou, Juan Mao, Baihong Chen

**Affiliations:** The College of Horticulture, Gansu Agricultural University, Lanzhou 730070, China; bianzy@gsau.edu.cn (Z.B.); 18394512517@163.com (Y.C.); luyang23zxc@163.com (Y.L.); liwenf@gsau.edu.cn (W.L.); lixin@gsau.edu.cn (X.L.); houyj@gsau.edu.cn (Y.H.); maojuan@gsau.edu.cn (J.M.)

**Keywords:** green manure, soil microbial community, fruit quality, Loess Plateau

## Abstract

Long-term clean tillage in apple orchards on the Loess Plateau has degraded soil properties and diminished fruit quality, highlighting the urgent need for sustainable cover cropping strategies. In a two-year field experiment (two consecutive growing seasons), eight green manure species (annual ryegrass, perennial ryegrass, white clover, smooth vetch, common vetch, rapeseed, hairy vetch, and cocksfoot) were compared with a clean-tillage control. Each treatment was replicated three times, and each plot was 100 m^2^. Soil physicochemical properties, microbial communities (16S rRNA and ITS amplicon sequencing), and fruit quality parameters were systematically assessed. All grass treatments significantly lowered soil pH and increased soil organic matter and total nitrogen, phosphorus, and potassium contents. Hairy vetch (T7) produced the highest soil organic matter (25.86 g kg^−1^) and total nitrogen (1.43 g kg^−1^), while rapeseed (T6) reduced pH most markedly (4.28%). White clover (T3) enhanced bacterial and fungal alpha-diversity and enriched beneficial genera (Sphingomonas, Lysobacter, Mortierella, Chaetomium). However, functional predictions based on 16S/ITS data using FAPROTAX and Tax4Fun (PICRUSt-type predictive tools) suggested a potential increase in chemoorganoheterotrophy and saprotrophy; these predicted functions should be interpreted as hypothesis-generating rather than as direct evidence of microbial function. Common vetch (T5) increased soluble sugar content by 28.1%, and cocksfoot (T8) decreased titratable acidity by 30.8%. The comprehensive analysis indicates that hairy vetch and white clover as the most effective species for improving soil fertility, microbial diversity, and fruit quality. We therefore recommend these two legumes as optimal cover crops for sustainable orchard floor management in rain-fed semi-arid regions of the Loess Plateau.

## 1. Introduction

Soil serves as the fundamental medium for agricultural production, with its nutrient content, structural integrity, and ecological condition directly influencing crop yield and the sustainability of agroecosystems [1]. Within conventional intensive cultivation systems, the excessive application of fertilizers has precipitated numerous issues, including soil acidification, compaction, depletion of organic matter, nutrient imbalances, and non-point source pollution. These factors have significantly compromised soil structure and functional stability, thereby posing constraints on food security and ecological safety [2]. Prominent agricultural regions such as the Taihu Basin, lychee-producing areas in southern China, and dryland farming zones in the northwest are confronted with shared challenges, including soil quality degradation, low efficiency in the utilization of nitrogen, phosphorus, and potassium, as well as deterioration of the soil microbial environment [3,4]. Prolonged continuous cropping and winter fallowing further accelerate fertility loss and reduce the resilience of agroecosystems [5]. Adopting scientifically informed tillage and cover-cropping practices is therefore essential not only for improving soil physicochemical properties and nutrient cycling but also for enhancing erosion control, carbon sequestration, biodiversity conservation, and the overall sustainability of low-carbon agriculture [6].

In recent years, grass cultivation in orchards has attracted considerable research interest because it can simultaneously sustain soil fertility, improve nutrient retention, regulate the microecological environment, and support fruit production [7,8]. This cultivation system promotes soil organic matter accumulation and nutrient cycling [9], systematically ameliorates soil physical properties [10], restores orchard ecological balance, and concurrently enhances both fruit yield and quality, thereby reconciling economic profitability with ecological sustainability [11]. Unlike clean tillage, which relies on herbicides and frequent intertillage, grass cultivation provides continuous ground cover and can reduce soil erosion, especially on sloping orchard land. Traditional orchard management predominantly relies on clean tillage, herbicides, and intertillage, with production heavily dependent on chemical fertilizers [12]. Although clean cultivation may temporarily improve management efficiency, its long-term implementation can lead to soil structural deterioration, loss of biological diversity, reduced productivity, and declining fruit quality [13]. At present, orchard grass cultivation is relatively common in Europe and the United States, where it has been adopted as a routine management practice and has led to the development of an integrated “fruit tree–forage–livestock” cycling system [14,15]. In China, however, the area under grass cultivation in orchards remains insufficient, and it is especially rare in orchards on the Loess Plateau [11]. The semi-arid Loess Plateau features deep soil layers, abundant sunlight, dry air, and low pest and disease pressure, offering favorable natural conditions for producing high-quality apples [16,17]. Nevertheless, most of this region receives only 400–500 mm of annual precipitation and is characterized as a typical rain-fed semi-arid agricultural zone. The soils of the Loess Plateau are predominantly derived from wind-blown loess and are rich in silt, making them highly susceptible to both water and wind erosion, apple production here primarily follows a dryland farming model that combines plastic film mulching beneath the tree canopy with clean cultivation in the inter-row spaces [18]. Given the limited adoption of green manure in this region and the need to integrate soil health, microbial function, and fruit quality, a systematic comparison of green manure species under local orchard conditions is urgently needed.

Soil microbial communities, which form the core of the rhizosphere microecosystem, directly influence how efficiently plants allocate photosynthetic products and the soil’s capacity to sequester carbon by secreting extracellular enzymes and forming mycorrhizal networks [19,20]. The diversity index of these microbial communities is commonly used as a key measure to assess the functionality of the rhizosphere microecosystem and serves as an important indicator for evaluating soil nutrient transformation processes [21]. Research has demonstrated that planting grasses not only increases the abundance of soil microorganisms but that this effect is further enhanced with longer periods of artificial forage cultivation [22]. Specifically, planting orchard grass can significantly boost microbial biomass and activity [9]. Earlier studies have shown that hairy vetch (Vicia villosa Roth) promotes soil fungal activity and helps maintain a stable environment for soil enzymes derived from microbes [23]. Another investigation confirmed that interplanting white clover (*Trifolium repens*) in apple orchards significantly raises soil organic carbon levels [24]. Although previous studies have shown that orchard grass cover can increase microbial biomass and activity [9,10,22], and that leguminous species such as hairy vetch and white clover can specifically stimulate fungal activity or soil organic carbon accumulation [23,24], it remains unclear whether different green manure species consistently affect bacterial and fungal alpha-diversity in the same direction. Moreover, the links among green manure-induced changes in soil pH, organic matter, and nutrient availability, microbial community composition, and fruit quality have rarely been tested simultaneously in rain-fed apple orchards on the Loess Plateau. We therefore formulated three hypotheses: different green manure species will differentially affect soil pH, soil organic matter, and nutrient availability; these soil chemical changes will be associated with shifts in bacterial and fungal alpha-diversity and community composition; and changes in soil chemical and microbial properties will be linked to improvements in fruit quality.

By regulating soil moisture and temperature, planting orchard grass not only alters the soil’s physical and chemical properties but also has a profound impact on mineral nutrient cycling, microbial diversity, population dynamics, and related enzyme activities [10,25]. Grass planting notably reduces nitrogen loss, highlighting its nitrogen-fixing capabilities [26]. In apple orchards, grass cover significantly lowers soil temperature, decreases evaporation, and improves water retention [27]. Furthermore, studies show that grass cover can reduce nutrient loss from the soil and effectively increase the levels of soil organic matter (SOM), available phosphorus, alkali-hydrolyzable nitrogen, available potassium, total phosphorus (TP), and total potassium (TK) in orchards [28,29,30].

Planting grass in orchards can boost the metabolic activity of fruit trees and increase the photosynthetic rate of their leaves, which positively influences tree growth and fruit quality [31,32]. Research has shown that orchards with grass cover have fruits with higher soluble sugar content, lower titratable acid levels, and significantly increased yields [33]. In vineyards, grass cover can notably raise the soluble solids content of grapes, enhance their vitamin C and anthocyanin levels, and reduce total acidity, thereby improving both the quality and aroma of the grapes [34]. Additionally, the grass-cattle-fruit system improves grape health and yield by regulating microbial communities on leaves and fruits [35]. Studies indicate that allowing grass to grow in orchards significantly enhances fruit quality, with longer grass growth periods further improving fruit taste and quality [36,37].

**Figure 1:**
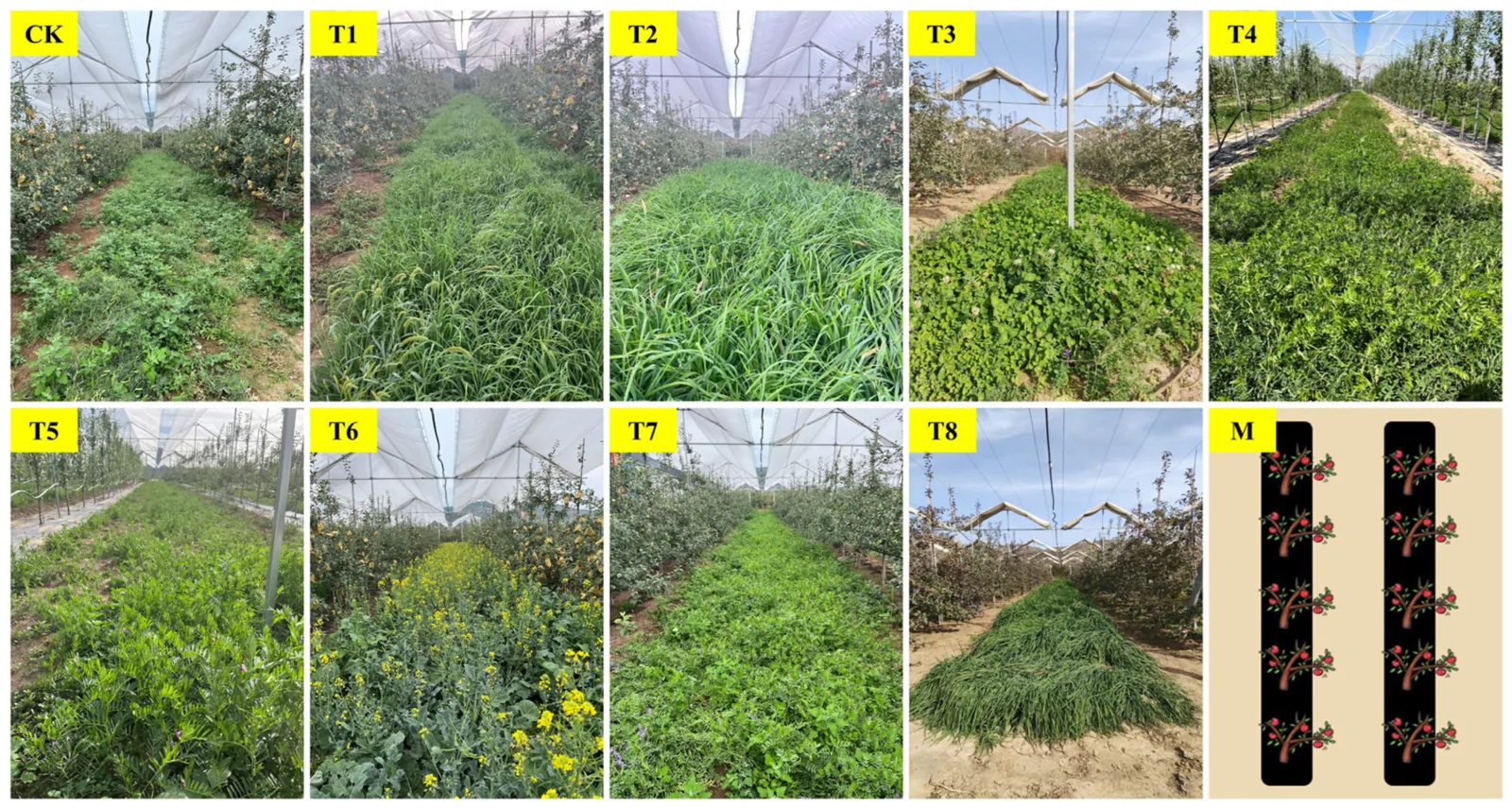
Pictures of the experimental site where green manure was planted. CK—clean tillage; T1—annual ryegrass; T2—perennial ryegrass; T3—white clover; T4—smooth vetch; T5—common vetch; T6—rapeseed; T7—hairy vetch; T8—cocksfoot; M—the cultivation model of apple orchards in the Loess Plateau (covering the soil under the trees with plastic film, carrying out inter-row tillage or planting green manure).

On the Loess Plateau, prolonged clear-tillage in orchards has degraded soil physical and chemical properties, negatively impacting fruit quality and yield. To test the above hypotheses, an experiment was conducted in an apple orchard located in Zhudian Town, Zhuanglang County, Pingliang City, Gansu Province (Figure 1). The study examined different types of cover crops planted between orchard rows, focusing on soil microbial diversity, soil physicochemical properties, mineral content, and fruit quality. Through correlation and principal component analyses, the study evaluated these factors comprehensively to determine the most suitable grass species for inter-row cover cropping in apple orchards. The goal of this research is to provide a scientific foundation for improving soil fertility, enhancing fruit quality, promoting sustainable apple orchard development, and advancing green cover cropping practices in orchards.

## 2. Results

### 2.1. The Effects of Grass Planting in Orchards on Soil Physical and Chemical Properties

Soil pH is an important factor influencing nutrient availability and the physical and chemical characteristics of soil. Although apple trees generally perform best in slightly acidic to neutral soils (pH 6.0–7.0) [38], they can grow and produce fruit in alkaline soils when adapted cultivars and rootstocks are used. In the calcareous soils of the Loess Plateau, where the native pH is typically above 8.0, a moderate reduction in pH may improve nutrient availability even if the soil remains above the optimal range. As indicated in Table 1, cover cropping significantly lowered soil pH compared to the control group (CK), though the extent of this reduction varied considerably among the different treatments. Specifically, the pH values for the eight green manure treatments ranged from 7.82 to 8.12, all of which were lower than the CK value (8.17 ± 0.01). Among these, treatment T6 had the lowest pH at 7.82, representing a significant 4.28% decrease compared to CK. This was followed by T2 with a pH of 7.99, which was 2.20% lower than CK. Treatments T3 and T7 had pH values of 8.04 and 8.00, corresponding to significant decreases of 1.59% and 2.08%, respectively. The pH values for T1 (8.10), T4 (8.11), T5 (8.09), and T8 (8.12) were also significantly lower than CK, though these reductions were relatively smaller at 0.86%, 0.73%, 0.98%, and 0.61%, respectively.

The impact of various treatments on soil organic matter (SOM) content exhibited statistically significant differences. Treatments T7 and T3 yielded the highest SOM contents, measuring 25.86 g/kg and 25.33 g/kg, respectively. No significant difference was observed between these two treatments; however, both were significantly greater than the other treatments, corresponding to increases of 34.07% and 31.36% relative to the CK, which had a SOM content of 19.29 g/kg. Treatments T2, T8, and T5 recorded SOM contents of 22.70 g/kg, 22.69 g/kg, and 22.25 g/kg, respectively, all sharing the same level of statistical significance and representing increases of 17.68%, 17.63%, and 15.35% compared to CK. Treatments T1 and T6 exhibited SOM contents of 21.88 g/kg and 20.66 g/kg, corresponding to increases of 13.43% and 7.10% relative to CK. Conversely, treatment T4 presented the lowest SOM content at 17.02 g/kg, indicating a significant decrease of 11.77% compared to the control.

With the exception of the T3 treatment, all other treatments significantly increased soil TN content. Treatment T7 had the highest TN content at 1.43 g/kg, a significant increase of 40.20% compared to CK (1.02 g/kg), representing the best improvement; treatments T2 and T8 had TN contents of 1.24 g/kg and 1.21 g/kg, respectively, with no significant difference between them, representing increases of 21.57% and 18.63% compared to CK; The TN contents in treatments T5, T1, T6, and T4 were 1.13 g/kg, 1.16 g/kg, 1.16 g/kg, and 1.10 g/kg, respectively, all at the same significance level, representing increases of 10.78%, 13.73%, 13.73%, and 7.84% compared to CK; The TN content in the T3 treatment was 1.08 g/kg, showing no significant difference from CK.

The effects of different treatments on soil TP content varied: some treatments significantly increased TP content, while others significantly decreased it. The T1 treatment had the highest TP content at 0.97 g/kg, representing a significant 34.72% increase compared to CK (0.72 g/kg), showing the most pronounced increase; The TP contents in treatments T5 and T7 were 0.84 g/kg and 0.83 g/kg, respectively, with no significant difference between them; both were 16.67% and 15.28% higher than CK, respectively. The TP contents in treatments T3, T4, and T8 were 0.74 g/kg, 0.81 g/kg, and 0.81 g/kg, respectively, with no significant difference from CK; The TP content in treatment T2 was 0.71 g/kg, showing no significant difference from CK; treatment T6 had the lowest TP content at 0.62 g/kg, representing a significant decrease of 13.89% compared to CK—the largest reduction.

Most treatments significantly increased the TK content in the soil; only treatments T6 and T3 showed no significant difference from CK. Treatment T6 had the highest TK content at 20.85 g/kg, representing a significant increase of 79.50% compared to CK (11.61 g/kg), making it the most effective treatment; treatments T5, T4, T7, T1, T8 had TK contents of 18.53 g/kg, 18.07 g/kg, 17.77 g/kg, 17.35 g/kg, and 16.70 g/kg, respectively, all at the same significance level, representing increases of 59.60%, 55.64%, 53.06%, 49.44%, and 43.84% compared to CK. The TK contents in treatments T3 and T6 were 14.78 g/kg and 13.37 g/kg, respectively, showing no significant difference from CK.

In summary, there were significant differences in the effects of different treatments on soil physicochemical properties: Treatment T6 had the most significant effect on lowering soil pH, but it also significantly reduced soil TP content; Treatment T7 had the best effects on increasing soil SOM and TN contents, while also significantly increasing TP and TK contents, demonstrating outstanding overall improvement; Treatment T2 had the most significant effect on increasing soil TK content, while also significantly increasing SOM and TN contents; Treatment T1 had the best effect on increasing soil TP content and also significantly increased SOM, TN, and TK levels; Treatment T4 significantly reduced soil SOM content and had a relatively poor overall improvement effect.

### 2.2. Soil Bacterial and Fungal Taxa

**Figure 2:**
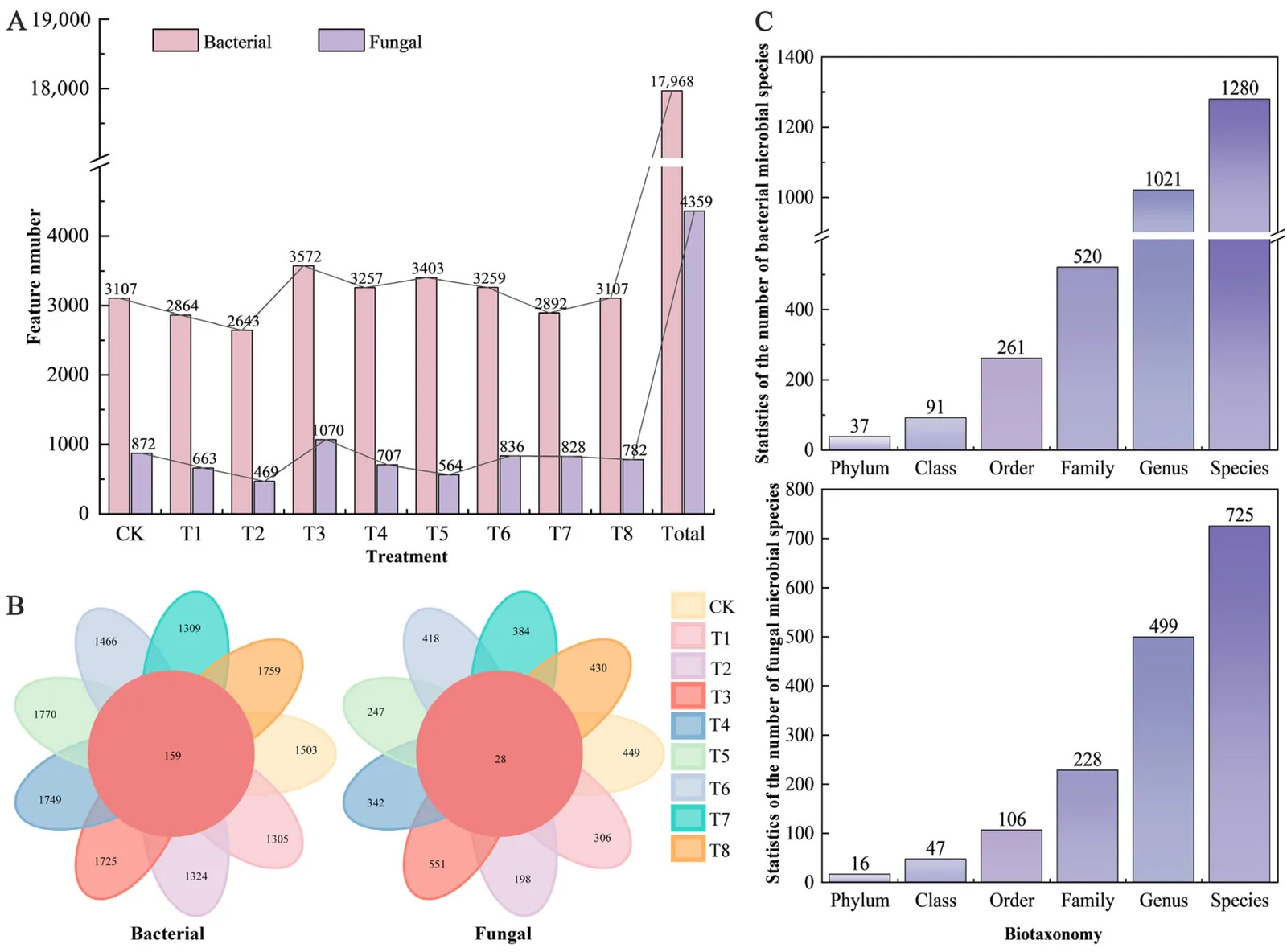
Statistical chart of the number of soil microbial OTUs under different grass types. (**A**) Distribution of OTUs of soil microorganisms under different grass types. (**B**) Venn diagram of soil microbial OTU number under different grass types. (**C**) Statistics of soil microbial species number under different grass types. CK, clean tillage; T1–T8 as defined in Figure 1. Bars in (**A**) represent OTU counts; error bars are not shown because sequencing was performed on composite samples per treatment.

An analysis of soil microbial diversity under different cover crop types in an apple orchard revealed that the total number of bacterial OTUs (Operational Taxonomic Units) across all cover crop types was 17,968, and the total number of fungal OTUs was 4359. Among these, treatment T3 had the highest number of microbial OTUs, with 3572 bacterial OTUs and 1070 fungal OTUs (Figure 2A). The nine sample groups shared a total of 159 bacterial OTUs, with treatment T5 having the highest number of unique OTUs at 1770; treatment T1 had the fewest, at 1305. The nine sample groups shared only 28 fungal OTUs, with treatment T3 having the highest number of unique fungal OTUs at 551; treatment T2 had the fewest, at 198. These results also reflect significant differences in soil bacterial and fungal microbial communities under different types of natural grass (Figure 2B, Table S1). Soil microbial classification shows that bacterial species in soil samples from different treatments primarily belong to 37 phyla, 91 classes, 261 orders, 520 families, 1021 genera, and 1280 species, while fungal species primarily belong to 16 phyla, 47 classes, 106 orders, 228 families, 449 genera, and 725 species (Figure 2C).

**Figure 3:**
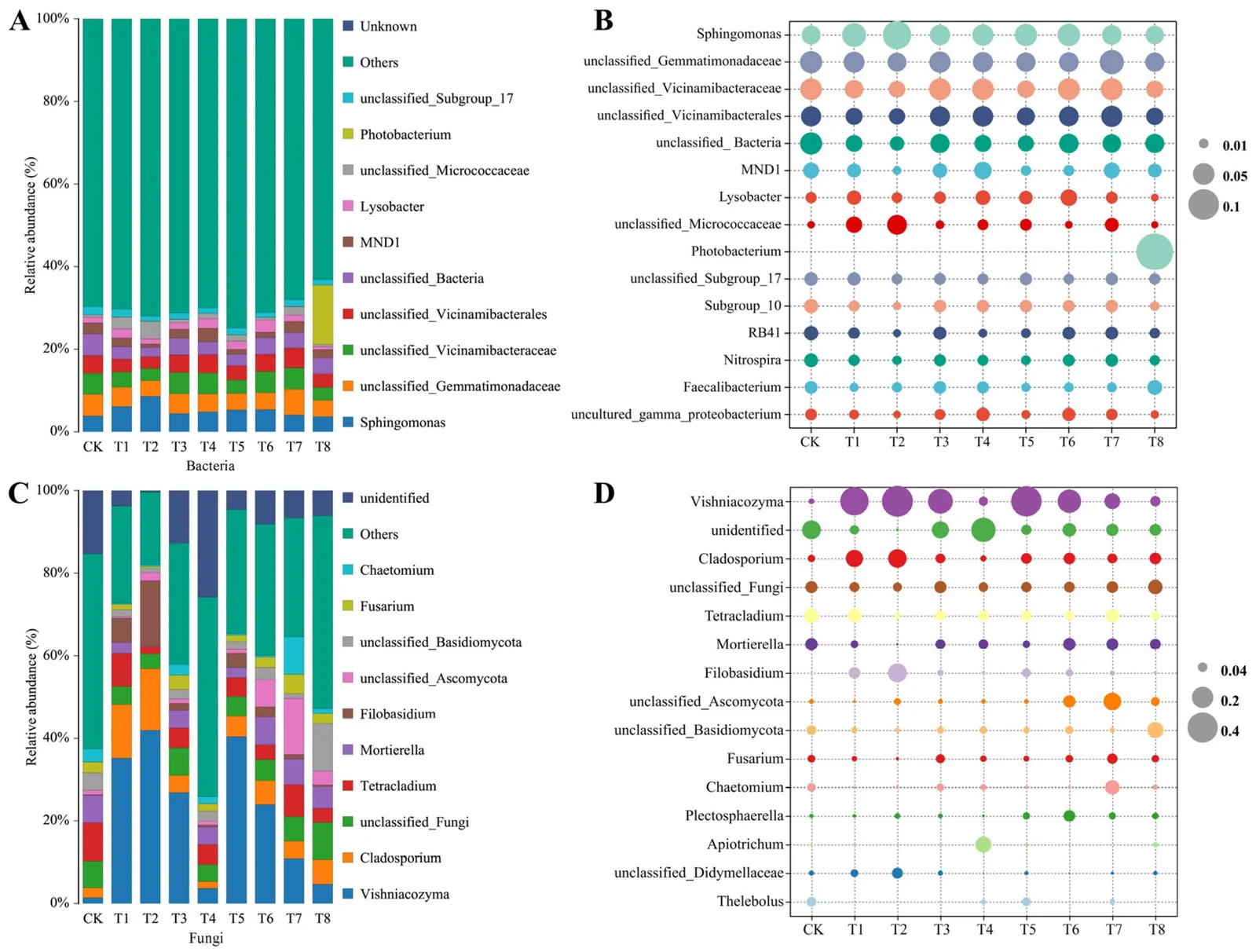
Map of the species abundance distribution at the genus level under different grass types. The relative abundances of the first 10 soil bacteria (**A**) and fungi (**C**) at the genus level, as well as the differences among the first 15 genera-level bacteria (**B**) and the differences at the phylum level among fungi (**D**) Bars represent the relative abundance (%) of each genus. “Others” includes all genera with relative abundance below the top 10, and “Unclassified” includes sequences without taxonomic annotation. CK, clean tillage; T1–T8 as defined in Figure 1. Data are based on 16S rRNA and ITS amplicon sequencing, with three replicates per treatment. Detailed values are provided in Supplementary Table S2.

A distribution bar chart was plotted for the top 10 species by abundance at the genus level (Figure 3, Table S2). The results show that, across the nine soil sample groups, more than 70% of the species not included in the top 10 were grouped under “Others,” while species without taxonomic annotation accounted for approximately 20%. The remaining 10% was dominated by the genera Sphingomonas and Lysobacter, which were the predominant microbial communities across all nine soil samples. These genera exhibited higher abundances under the T2 and T6 treatments. These bacteria secrete extracellular hydrolases, produce a diverse range of antimicrobial secondary metabolites, and degrade certain pesticides, thereby improving the soil environment. Consistent with the bacterial community, the top 10 fungal species in the samples, along with unidentified taxa and species without taxonomic annotation, accounted for a significant proportion. Compared to the CK group, the T3 and T7 treatments significantly increased the relative abundances of Vishniacozyma, Cladosporium, Filobasidium, Tetracladium, Mortierella, and Chaetomium, indicating that the dominant fungal genera under the T3 and T7 treatments were Chaetomium, Mortierella, and Cladosporium. These genera can assist plants in absorbing nutrients from the soil and promote plant growth.

### 2.3. Alpha and Beta Diversity of the Microbial Communities

The Shannon diversity index of bacterial communities across treatments ranged from 9.28 to 9.73 (Table 2, Table S3). Treatment T3 exhibited the highest bacterial Shannon index (9.73), which was significantly greater than that observed in T2 (9.28), while no significant differences were detected between T3 and the other treatments aside from T2. Regarding bacterial richness, as measured by the Chao1 index, values varied from 1159.46 (T2) to 1431.08 (T3). Treatment T3 demonstrated a significantly elevated bacterial Chao1 index compared to all other treatments (*p* < 0.05), followed by T5 (1365.20) and T6 (1332.83), both of which were significantly higher than CK, T1, T2, T4, T7, and T8. The lowest bacterial Chao1 value was recorded in T2, which was significantly lower than all treatments except T1.

For fungal communities, the Shannon index ranged from 4.77 (T2) to 6.78 (T3). Treatment T3 presented the highest fungal Shannon index (6.78), significantly surpassing all other treatments except T6 and T7. The fungal Chao1 index varied between 213.00 (T2) and 449.00 (T3), with T3 again exhibiting the highest value, significantly exceeding all other treatments (*p* < 0.05). Treatments T6 (352.84) and T7 (347.00) also showed relatively high fungal richness, significantly greater than T1, T2, T5, and T8. The lowest fungal Chao1 index was observed in T2 (213.00), which did not differ significantly from T1, T5, or T8, but was significantly lower than CK, T3, T4, T6, and T7.

In summary, treatment T3 consistently enhanced alpha-diversity in both bacterial and fungal communities, as indicated by the highest Shannon and Chao1 indices across these microbial groups. In contrast, treatment T2 generally suppressed microbial diversity and richness, exhibiting the lowest values for both bacterial and fungal Shannon and Chao1 indices. These findings suggest that T3 positively influenced the complexity of soil microbial communities, whereas T2 had a detrimental effect on microbial community structure.

**Figure 4:**
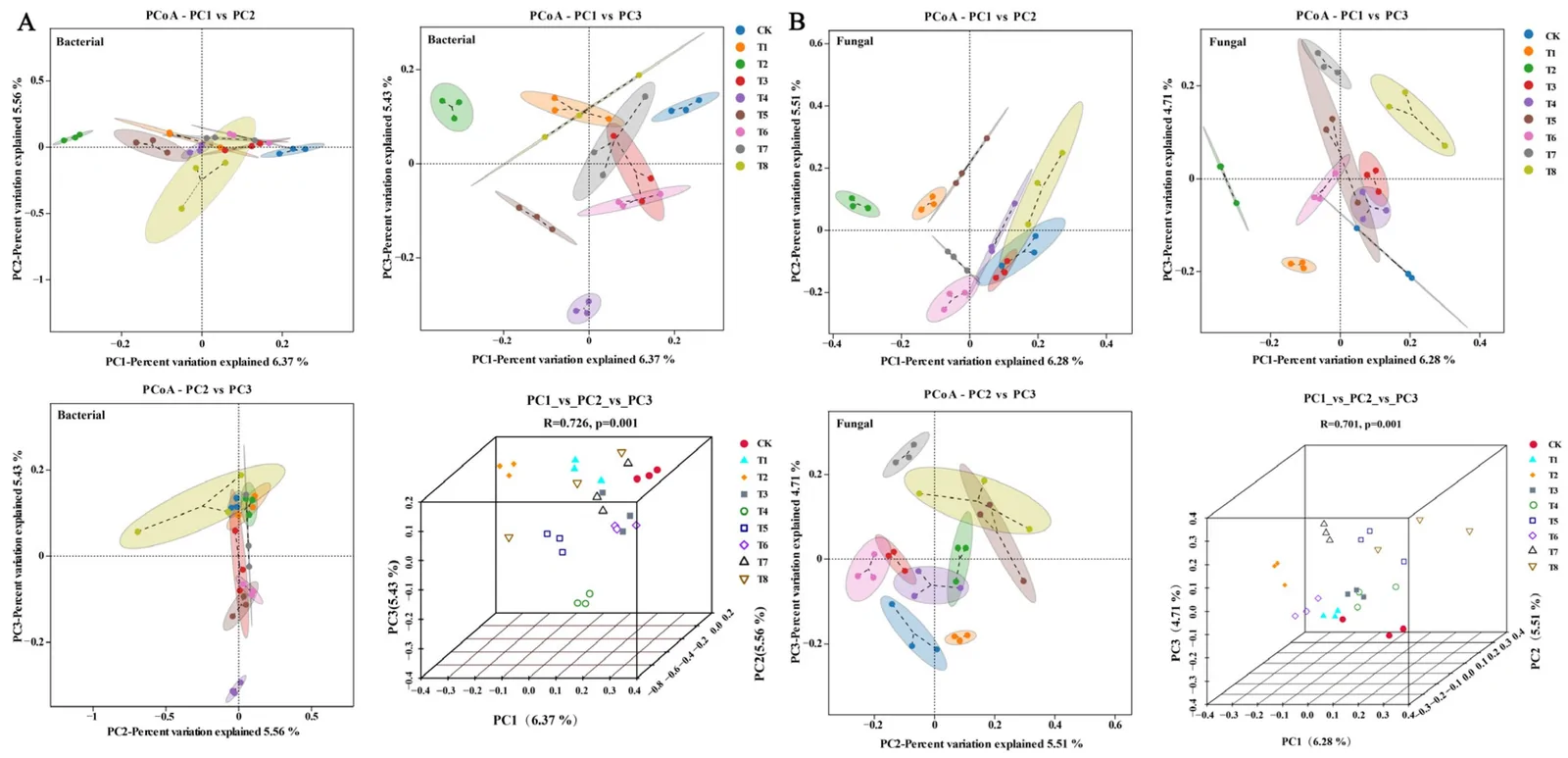
Principal coordinate analysis (PCoA) of soil microbial communities under different green manure treatments. (**A**) Bacterial communities. (**B**) Fungal communities. PCoA was based on Bray–Curtis distances. Each point represents one sample (*n* = 3 per treatment). Ellipses represent 95% confidence intervals. The percentages on the axes indicate the variance explained by each principal coordinate. CK, clean tillage; T1–T8 as defined in Figure 1. Differences in community composition among treatments were tested by PERMANOVA, and the relationship between soil physicochemical properties and community composition was assessed by Mantel test (Figure S1).

Based on the Bray–Curtis distance, a principal co-ordinates analysis (PCoA) was conducted to analyze the soil microbial Beta diversity (Figure 4, Table S4). As depicted in Figure 4A, the first principal coordinate (PC1) accounts for 6.37% of the variation observed among bacterial communities, while the second (PC2) and third (PC3) principal coordinates explain 5.56% and 4.35% of the variation, respectively. Compared to the CK, the triplicate samples within the T1, T7, and T8 treatment groups exhibited greater dispersion. Similarly, the principal coordinate analysis (PCoA) of fungal communities, shown in Figure 4B, indicated that PC1 explained 6.28% of the variation among groups, with PC2 and PC3 accounting for 5.51% and 4.71%, respectively. These results indicate that the treatments exerted significant effects on microbial community composition, with bacterial communities demonstrating a marginally more distinct separation along the primary ordination axes relative to fungal communities. Mantel test was performed to elucidate the linkages between soil physicochemical properties and microbial community composition (Figure S1). Among all measured variables, soil pH showed the strongest correlation with bacterial community structure (r = 0.69, *p* = 0.039), indicating that pH was the predominant driver shaping bacterial β-diversity.

### 2.4. Bacterial and Fungal Ecological Functions

**Figure 5:**
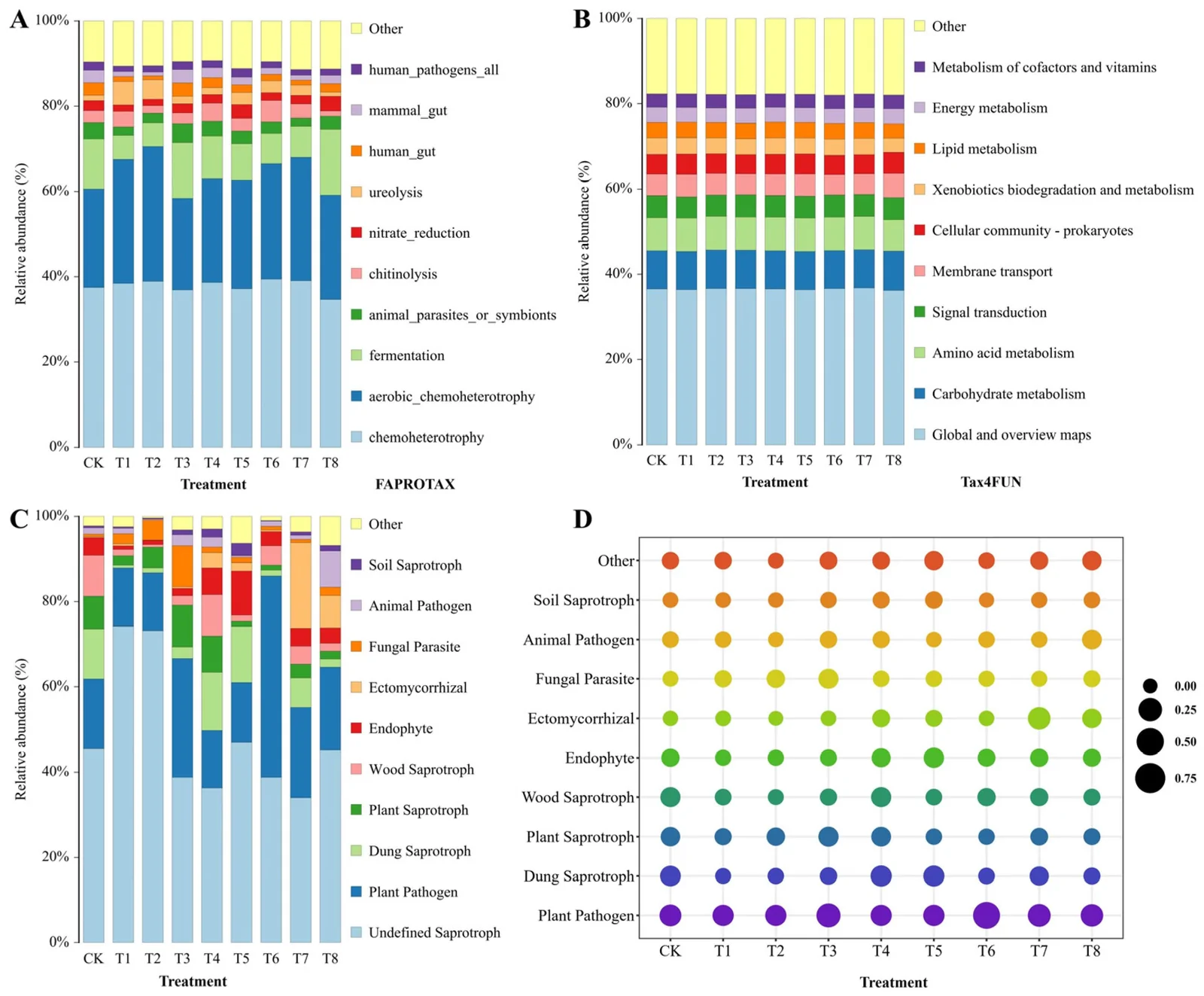
Predicted ecological functions of soil microbial communities under different green manure treatments. (**A**) FAPROTAX-predicted bacterial functions; (**B**) Tax4Fun-predicted KEGG level 2 metabolic pathways; (**C**) FUNGuild-predicted fungal trophic modes; (**D**) bubble plot summarizing the relative abundance of fungal functional guilds. Relative abundances are expressed as percentages. CK, clean tillage; T1–T8 as defined in Figure 1. These functional profiles are predictions inferred from taxonomic composition and do not directly measure gene expression, enzyme activity or metabolic fluxes; they should therefore be interpreted as hypothesis-generating rather than as direct functional evidence. No statistically significant differences among treatments were detected for Tax4Fun pathways (*p* > 0.05).

To explore the potential functional consequences of the observed alterations in microbial community composition, we conducted predictive functional profiling utilizing FAPROTAX (version 1.2.4) for bacterial taxa and Tax4FUN (v1.0) for metabolic pathway prediction (Figure 5, Table S5). It should be emphasized that FAPROTAX and Tax4Fun are predictive tools based on taxonomic composition and do not directly measure gene expression, enzyme activity, or actual metabolic fluxes. Therefore, examination of the FAPROTAX-predicted ecological functions for the ten most abundant bacterial species in the sampled soils suggested that bacterial functions in apple orchard soil were potentially associated with chemotrophic heterotrophy, oxo-chemotrophic heterotrophy, fermentation, and nitrate reduction. These results represent predicted functional potentials rather than directly measured activities. At the KEGG metabolic pathway level 2, Tax4FUN analysis predicted that the principal functional categories included carbohydrate metabolism, amino acid metabolism, signal transduction, membrane transport, lipid metabolism, and energy metabolism, with no statistically significant differences detected among the treatment groups. Given the limitations of predictive functional tools, these pathway-level results should be considered exploratory.

Comparative analysis of the relative abundances of soil fungal communities across different grass cultivation treatments suggested that undefined saprotrophs constituted over 70% of the fungal community in the T1 and T2 groups. In contrast, the T6 group exhibited a notably higher relative abundance of predicted plant pathogenic fungi, accounting for 47.2% (Figure 5C,D). The presence of grass was predicted to moderately enhance the relative abundances of soil saprotrophs, ectomycorrhizal fungi, and fungal parasites, while concurrently reducing the prevalence of wood saprotrophs within the soil microbial community. These fungal functional assignments are based on taxonomic inference and should be treated as putative rather than confirmed ecological functions.

### 2.5. The Effect of Green Manure Cultivation Model on Apple Fruit Quality

**Figure 6:**
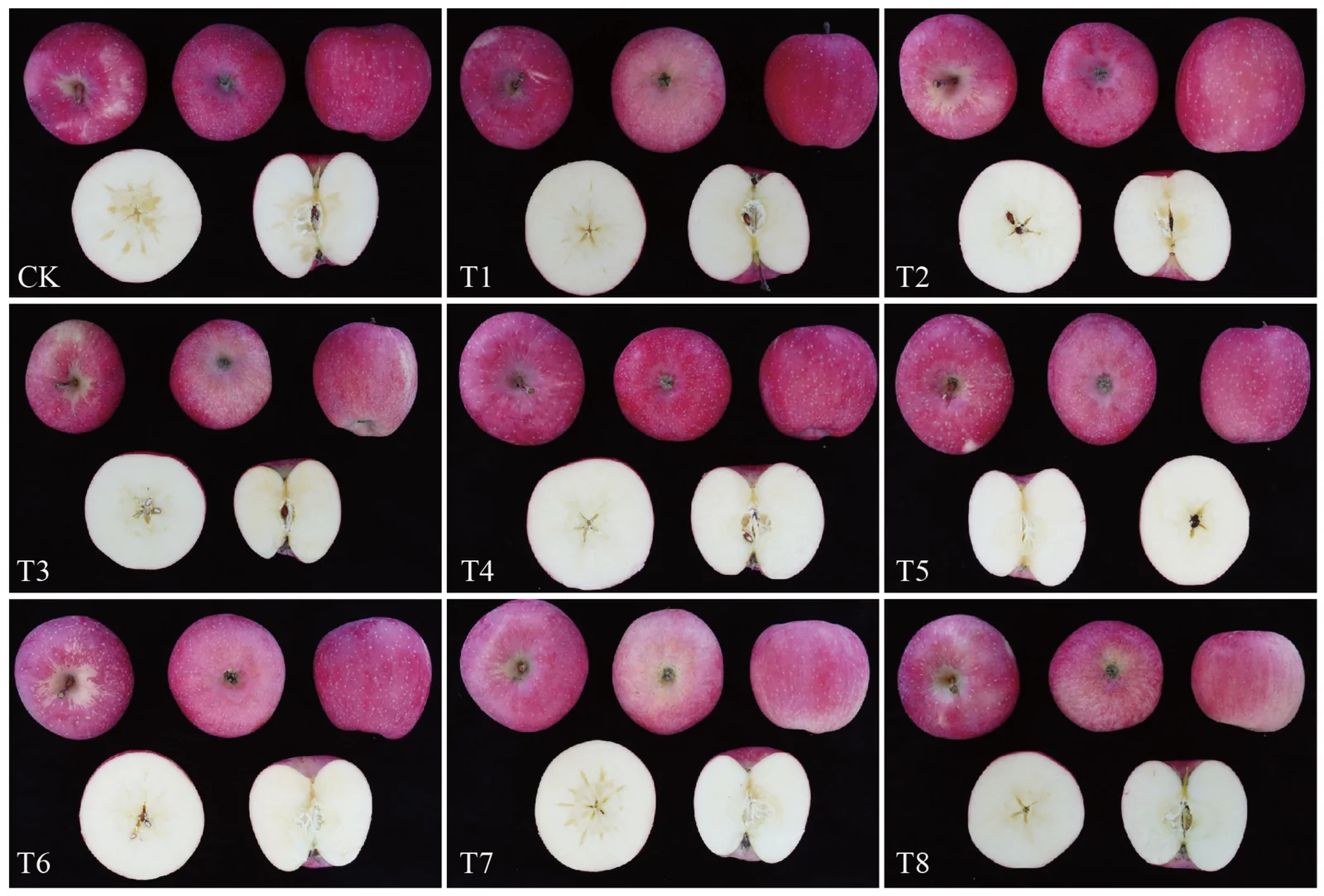
Appearance and cross-section view of the fruit of ‘Yanfu No.3’ under different green manure treatments. Photographs show representative fruits from each treatment. CK, clean tillage; T1–T8 as defined in Figure 1. Three biological replicates were used per treatment, and at least 30 fruits per treatment were examined for external quality analysis.

**Figure 7:**
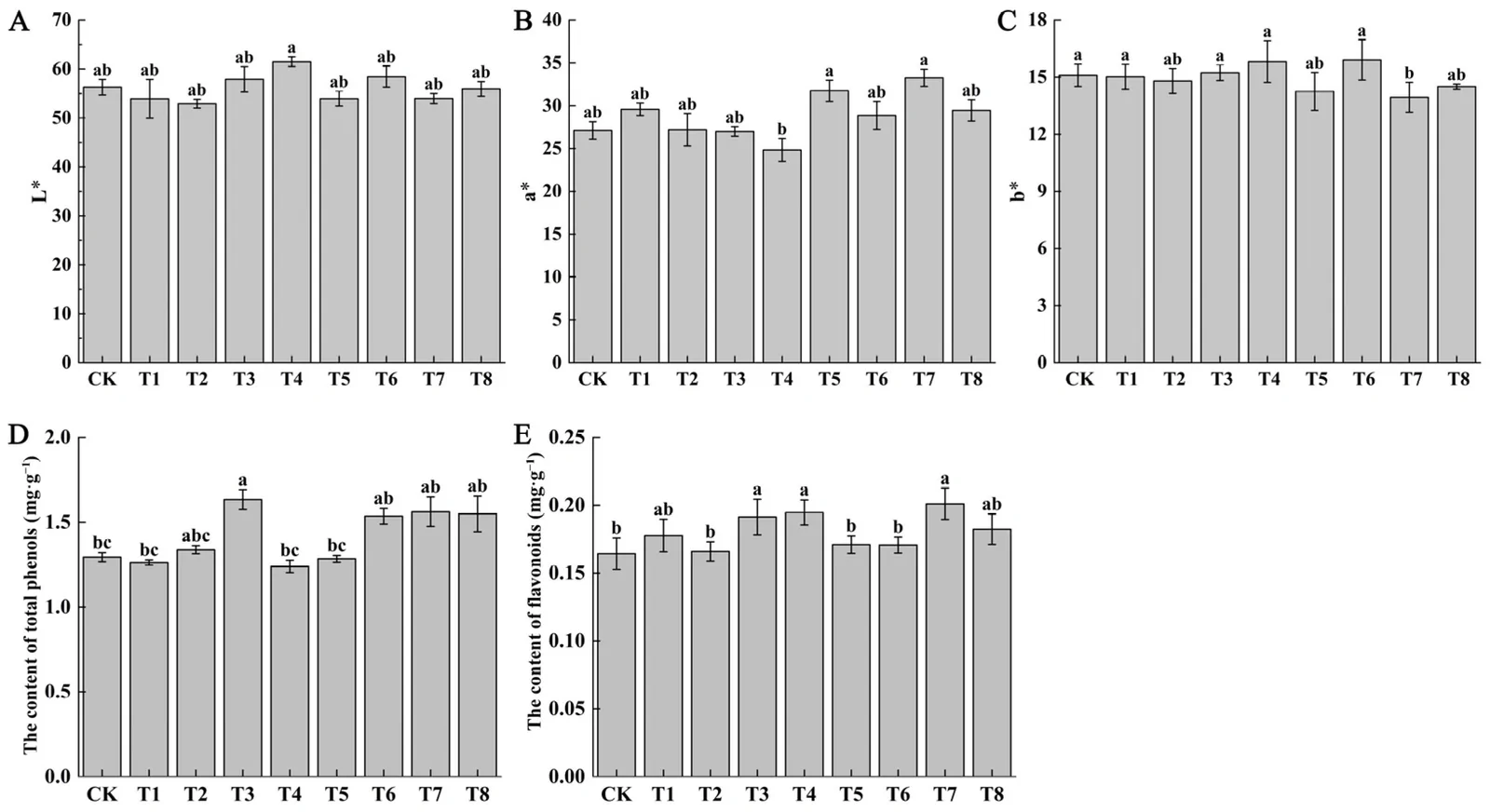
Effects of orchard grass planting on fruit peel color, total phenols and total flavonoids in ‘Yanfu No.3’ apple. (**A**–**C**) Skin color parameters L*, a* and b* (dimensionless). (**D**) Total phenol content (mg g^−1^). (**E**) Total flavonoid content (mg g^−1^). CK, clean tillage; T1–T8 as defined in Figure 1. Error bars represent mean ± SD (*n* = 3). Different lowercase letters indicate significant differences among treatments based on Tukey’s HSD test (*p* < 0.05).

The fruit phenotypes demonstrated that different grass species and treatment methods had a significant impact on the color differences in the fruits (Figure 6 and Figure 7). Treatments T3, T4, and T6 notably increased the L value to 57.91, 61.50, and 58.43, respectively, greatly enhancing the fruit’s appearance and color. In contrast, the L values of the fruit peel in treatments T1, T2, T5, and T7 were significantly lower than those in the control group. The a-values for treatments T5 and T7 were significantly higher than the control, while the a-value for treatment T4 was lower than the control. For the b-values, treatments T1 and T3 showed no significant difference compared to the control; treatments T4 and T6 had significantly higher b-values, whereas treatments T5, T7, and T8 had significantly lower b-values than the control.

**Figure 8:**
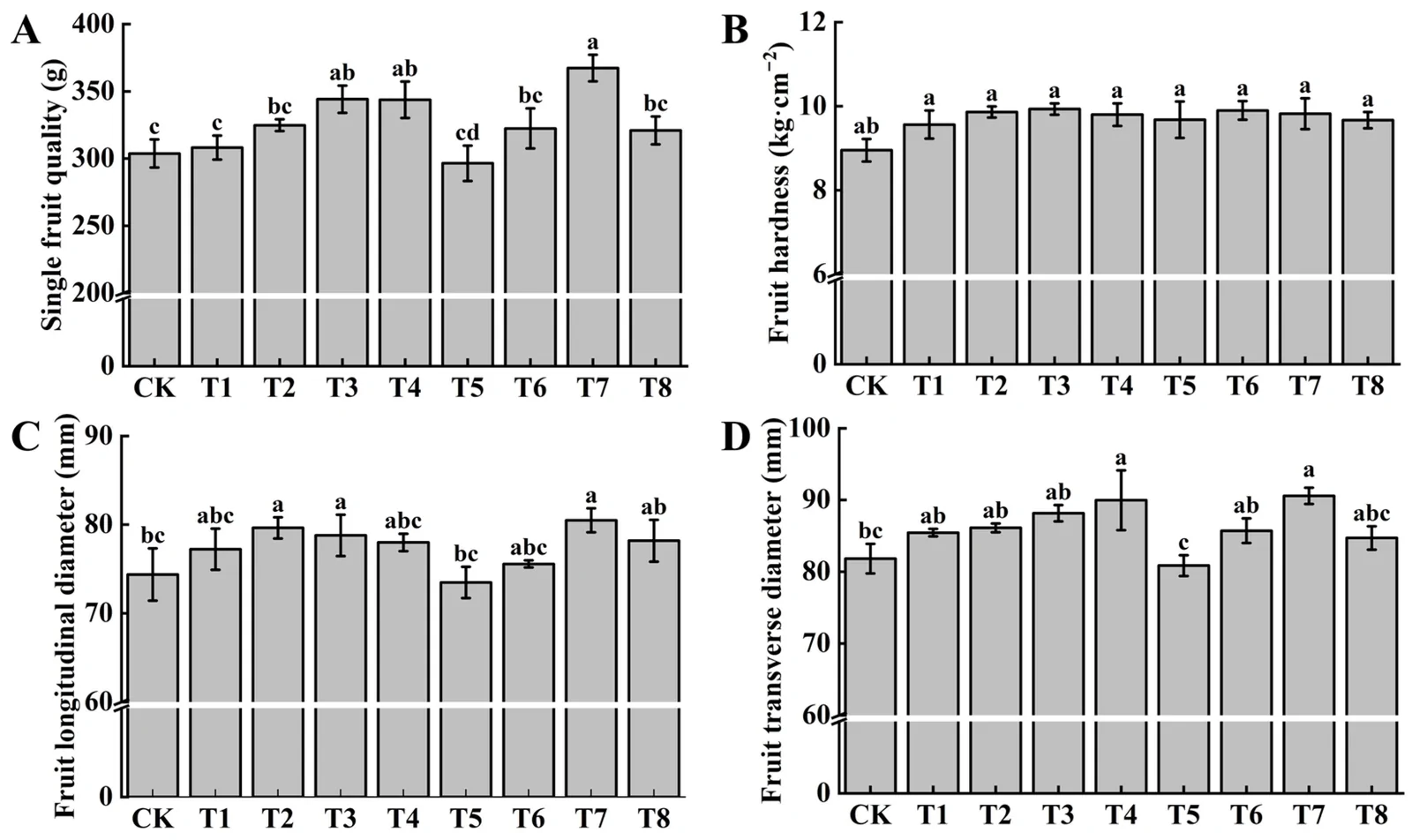
The influence of planting grass in orchards on the single fruit quality, hardness and length-width ratio of fruits. (**A**) single fruit quality; (**B**) fruit hardness; (**C**) fruit longitudinal diameter; (**D**) fruit transverse diameter. Error bars represent mean ± SD (*n* = 3). Different letters indicate significant differences (*p* < 0.05 by Duncan’s test).

An analysis of fruit appearance quality across different grass types showed that the individual fruit weights for treatments T3, T4, and T7 were 334.14 g, 333.69 g, and 367.36 g, respectively, which correspond to increases of 18.6%, 18.43%, and 30.38% compared to CK. Fruit firmness was highest under treatment T1, measuring 10.56 kg·cm^−2^, a 17.98% increase over CK; the other treatments showed no significant differences in firmness, with values ranging from 9.66 to 9.93 kg·cm-2. For the CK treatment, fruit length was 74.37 mm and diameter were 81.81 mm. Compared to CK, treatments T2, T3, and T7 significantly increased fruit length by 7.07%, 5.93%, and 8.22%, respectively. Treatment T7 produced the largest fruit diameter at 90.57 mm, a 10.71% increase over CK (Figure 8). Overall, treatment T7 notably enhanced individual fruit weight.

**Figure 9:**
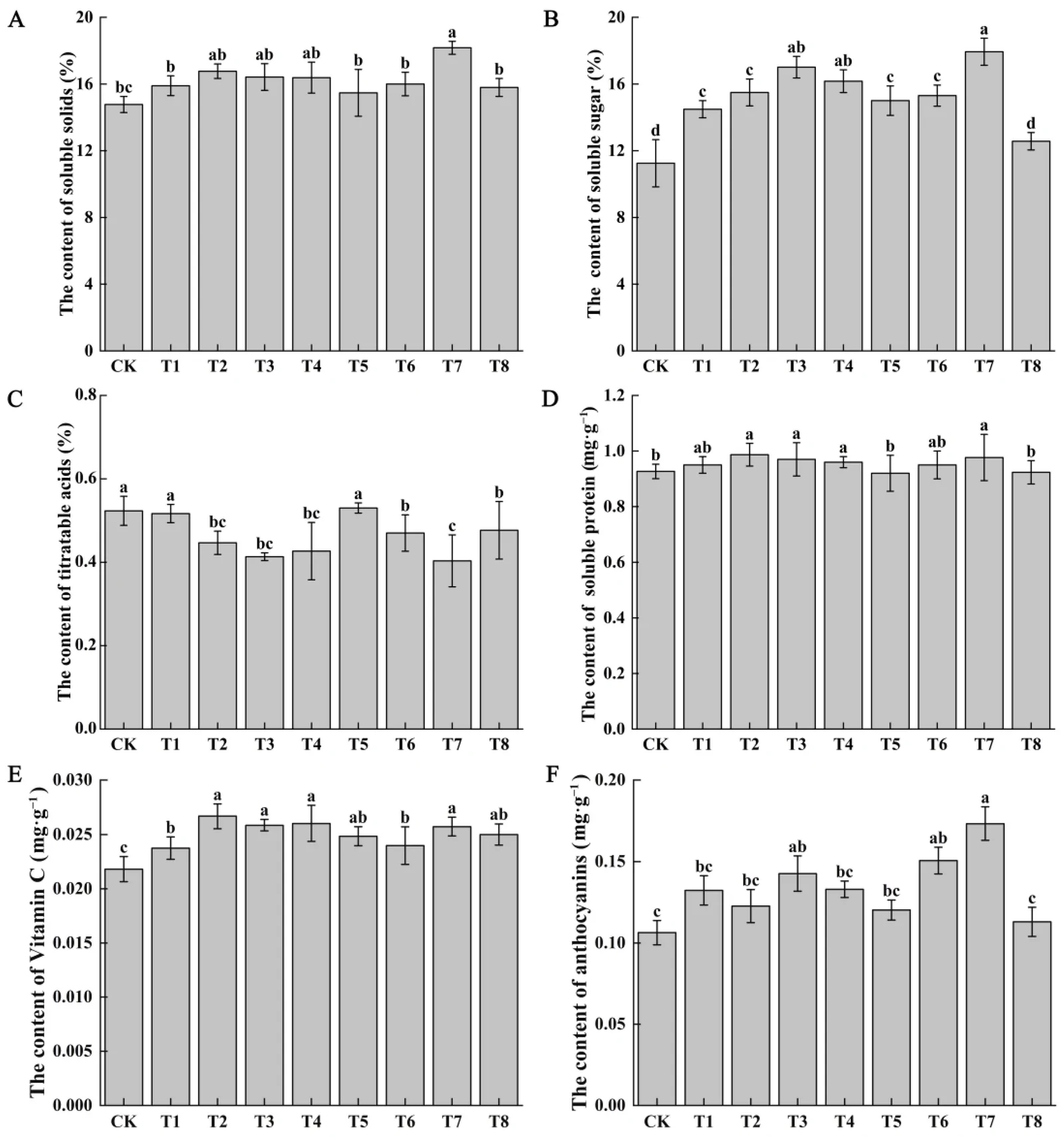
The influence of planting grass in orchards on the internal quality of fruits. (**A**) The content of soluble solids. (**B**) The content of soluble sugar. (**C**) The content of titratable acids. (**D**) The content of soluble protein. (**E**) The content of Vitamin C. (**F**) The content of anthocyanins. Error bars represent mean ± SD (*n* = 3). Different letters indicate significant differences (*p* < 0.05 by Duncan’s test).

An analysis of soluble solids content in mature fruits showed that, compared to the CK, all grass-cover treatments increased the fruit’s soluble solids content to varying extents, with the T7 treatment producing the most notable increase. Under T7, the soluble solids content in the fruit reached up to 18.17%. The differences in soluble solids content among the other treatments were not significant, averaging 16.09%, but all were still higher than the control (Figure 9A).

Regarding soluble sugar content, fruits grown under the different grass-cover treatments generally had higher levels than those under clean tillage. The soluble sugar content ranked as follows: T7 (17.93%) > T3 (17.00%) > T4 (16.16%) > T2 (15.49%) > T6 (15.30%) > T5 (15.00%) > T1 (14.49%) > T8 (12.57%) > CK (11.25%). The soluble sugar content in fruits from the T3, T4, and T7 treatments was significantly higher than that of the control. While there were no significant differences among the T1, T2, T5, and T6 treatments, all still had higher soluble sugar content than the control (Figure 9B). It should be noted that these results represent compositional changes in fruit quality. Marketable yield (e.g., yield per tree or per hectare, fruit grade, and marketable fruit rate) was not measured in this study; therefore, we cannot conclude that the observed increases in soluble sugar or reductions in acidity directly translated into higher marketable yield.

Except for treatment T5, all other treatments decreased the titratable acid content in the fruit. The lowest titratable acid content was observed under treatment T7 at 0.40%, which is a significant 23.08% reduction compared to the CK at 0.52%. Treatments T2, T3, and T4 followed in terms of reduction. In contrast, treatment T5 resulted in a higher titratable acid content than the control, measuring 0.53%. There was no significant difference in titratable acid content between treatments T6 and T8. Although common vetch (T5) increased soluble sugar content and cocksfoot (T8) reduced titratable acidity, these effects were observed at the level of fruit composition rather than marketable yield. Regarding fruit soluble protein content, variations were noted among the different orchard grassing treatments. Treatments T2, T3, T4, and T7 significantly increased the soluble protein content compared to the control (Figure 9C). Treatment T2 had the highest soluble protein content at 0.99 mg·g^−1^, representing a 7.92% increase over the control’s 0.93 mg·g^−1^. Treatments T1 and T6 also raised soluble protein levels, but there was no significant difference between them, both measuring 0.95 mg·g^−1^. Treatments T5 and T8 showed no significant difference from the control in soluble protein content (Figure 9D).

The analysis of fruit vitamin C (Vc) content under different grass treatments showed that the Vc levels in treatments T2, T3, T4, and T7 were significantly higher than in the control group (CK), averaging 0.026 mg·g^−1^. This represents an 18.18% increase compared to the CK value of 0.022 mg·g^−1^. In treatments T5 and T8, the fruit Vc content was 0.025 mg·g^−1^, while treatments T1 and T6 had a Vc content of 0.024 mg·g^−1^ (Figure 9E). Regarding anthocyanin content in the fruit peel, the highest level was observed in treatment T7 at 0.173 mg·g^−1^ (Figure 9F). Other treatments also enhanced anthocyanin content to varying extents, ranked from highest to lowest as follows: T6 (0.150 mg·g^−1^), T3 (0.142 mg·g^−1^), T4 (0.133 mg·g^−1^), T1 (0.132 mg·g^−1^), T2 (0.122 mg·g^−1^), T5 (0.120 mg·g^−1^), and T8 (0.113 mg·g^−1^).

In summary, treatments T2, T3, T4, and T7 influenced the fruit’s soluble solids, soluble sugars, and titratable acids by increasing soluble solids and sugars while decreasing titratable acids. T3 and T7 can enhance the vitamin C content in the fruits and the total anthocyanin content in the fruit skins.

## 3. Discussion

### 3.1. Shifts in Soil Microbial Community Structure and Ecological Functions Induced by Green Manure Species

**Figure 10:**
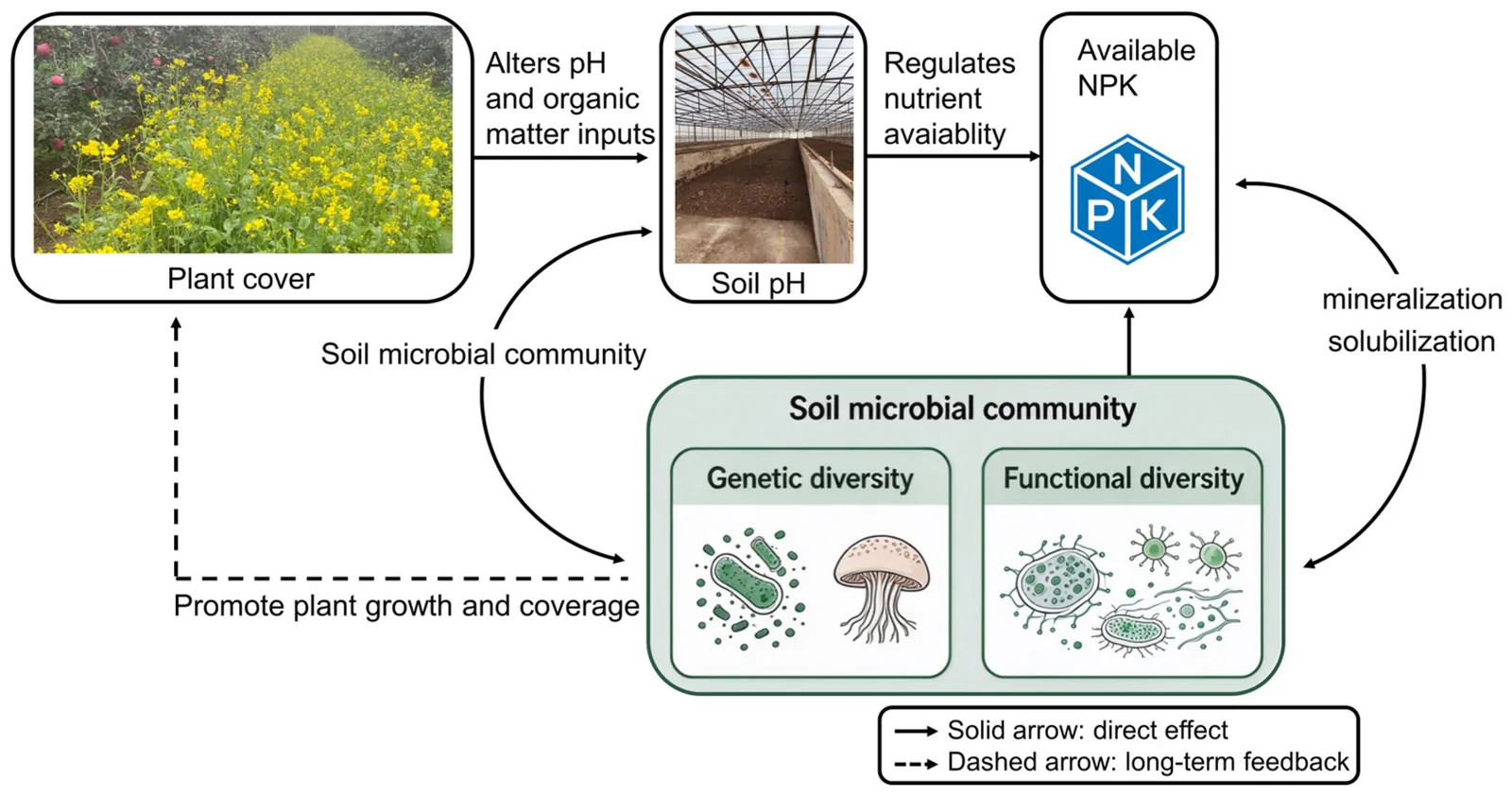
Conceptual framework linking green manure cover, soil chemical properties, microbial communities and nutrient availability in apple orchards.

To better interpret the relationships among the measured variables, we summarized the proposed interactions in a conceptual diagram (Figure 10). As shown, green manure cover can modify soil pH and organic matter, which in turn regulate total nutrients and microbial community structure. The microbial community is not merely a passive responder; it actively feeds back on nutrient availability by producing extracellular enzymes, organic acids, and symbiotic structures that mineralize organic matter, solubilize phosphorus, and enhance nitrogen uptake. This feedback loop provides a mechanistic link between the soil chemical changes and the fruit quality improvements observed in this study. Soil microbial communities are sensitive bioindicators of management practices, and their composition directly influences nutrient cycling and soil health. In this study, inter-row grass planting significantly altered both bacterial and fungal community structures, with white clover (T3) showing the highest alpha-diversity indices. The dominance of Sphingomonas and Lysobacter in T2 and T6 treatments is consistent with previous reports that these taxa secrete extracellular hydrolases and antimicrobial metabolites, thereby suppressing pathogens and degrading recalcitrant compounds [39]. Fungal communities under T3 and T7 were enriched with Vishniacozyma, Cladosporium, and Mortierella, which are known to facilitate phosphorus solubilization and mycorrhizal-like nutrient transfer [40]. Although functional profiling via FAPROTAX and Tax4Fun suggested that grass cover may promote chemoorganoheterotrophy and carbohydrate/amino acid metabolism, these results are inferential and should be interpreted cautiously because such PICRUSt-type tools rely solely on taxonomic composition and cannot capture gene expression, enzyme activity, or functional redundancy. The predicted increase in saprotrophs and ectomycorrhizal fungi, along with reduced wood saprotrophs, is consistent with, but does not provide direct evidence for, a shift towards more efficient decomposition and symbiotic nutrient acquisition. Future metagenomic, metatranscriptomic, or culture-based studies are needed to validate these predicted functions. These results corroborate findings from meta-analyses showing that grass cover increases microbial biomass and activity, and that leguminous species exert stronger selection effects on community composition than gramineous ones [41,42]. The observed treatment-specific modulation of microbial guilds underscores the importance of choosing appropriate grass species to steer soil functions toward desired outcomes, such as carbon sequestration or disease suppression.

### 3.2. Green Manure Effects on Soil Physicochemical Properties and Nutrient Availability

The significant reduction in soil pH under all grass treatments, especially rapeseed (T6), can be attributed to root exudation of organic acids and the release of CO_2_ and acidic compounds during residue decomposition, which activates mineral nutrients and improves their availability [43,44,45]. The marked increases in SOM and TN under hairy vetch (T7) and white clover (T3) reflect the high biomass production and biological nitrogen fixation capacity of legumes, which enhance carbon input and nitrogen accumulation in the topsoil. Notably, the total phosphorus (TP) and total potassium (TK) responses varied among treatments: T1 (annual ryegrass) gave the highest TP increase (34.7%), while T2 (perennial ryegrass) maximized TK (79.5% increase), indicating species-specific mobilization of different elements. This differential effect may arise from variations in root morphology, exudate chemistry, and associated microbial communities that mediate mineral weathering and solubilization. Collectively, these changes enhance the soil’s capacity to retain water and nutrients, which is particularly critical in the semi-arid Loess Plateau where rainfall is limited and erratic. The results highlight that a combination of legume and grass species could offer balanced nutrient management, but for practical recommendations, hairy vetch and white clover stand out as the most effective single-species options for overall soil fertility enhancement in this region.

### 3.3. Implications for Fruit Quality Improvement and Sustainable Orchard Management

The beneficial effects of grass cover on apple fruit quality are demonstrated by increased soluble sugar levels and decreased titratable acidity under certain treatments. Specifically, common vetch (T5) and smooth vetch (T4) were especially effective at raising soluble sugar content, while cocksfoot (T8) and hairy vetch (T7) lowered acidity and improved the sugar–acid balance, which is crucial for consumer preference. These enhancements likely result from two interconnected processes: (i) improved soil nitrogen availability from legume residues boosts photosynthetic carbon assimilation and sugar allocation [46,47], and (ii) changes in soil microbial communities, particularly the increase in plant growth-promoting fungi and bacteria, may enhance root hormone signaling and carbohydrate metabolism [48,49]. However, it is important to emphasize that these improvements were assessed as compositional quality traits rather than as marketable yield. Because fruit yield, fruit grade, and marketable fruit rate were not quantified in this study, the practical question of whether these quality shifts translate into higher economic returns remains open. In addition, the single-fruit weight of T7 was significantly higher than that of CK (Figure 8A), suggesting a possible yield advantage, but this cannot substitute for direct yield measurements. Future studies should therefore include marketable yield, fruit grading, and economic benefit analysis to fully evaluate the practical value of these green manure treatments. Although some treatments successfully reduced soil pH, they also decreased total phosphorus content, indicating a trade-off that needs consideration. Moreover, several additional trade-offs should be considered before widely recommending hairy vetch and white clover. Hairy vetch produces high biomass and has strong water uptake, which may intensify soil water depletion in the rain-fed semi-arid Loess Plateau, especially in dry years or in orchards without supplemental irrigation. White clover is a perennial legume that can persist for multiple years, but its continuous cover may compete with apple trees for water and nutrients, and it may require more frequent mowing or management to prevent excessive growth. In addition, both species may serve as alternative hosts for certain pests or pathogens, although no obvious disease problems were observed in this study. The optimal management strategy may therefore involve a mixture of legume and grass species to balance nitrogen fixation, water use, and ground cover, rather than relying on a single species. Long-term monitoring of soil water dynamics, tree growth, and yield is needed to confirm the sustainability of these recommendations. Overall, hairy vetch and white clover consistently performed best in terms of soil fertility, microbial diversity, and fruit quality. Thus, these two species are recommended as the best cover crops for apple orchards on the Loess Plateau. Their use can lower dependence on chemical fertilizers, prevent soil degradation, and improve both the ecological and economic sustainability of apple farming in rain-fed dryland areas. Future studies should examine long-term impacts over several growth cycles and assess mixed-species plantings to further enhance beneficial interactions.

This study has several limitations. First, we measured total nutrient pools (TN, TP, TK) rather than plant-available fractions (alkali-hydrolyzable N, available P, available K). Total nutrient pools represent long-term reservoirs and may not directly reflect nutrient availability to plants and microorganisms. Therefore, we cannot directly evaluate the relationships between available nutrients, microbial diversity, and fruit quality. Future studies should include measurements of available nutrient fractions to clarify these relationships. Second, marketable yield was not measured, and the fruit quality results should be interpreted as compositional changes rather than confirmed yield gains.

## 4. Materials and Methods

### 4.1. Study Site Description

The experiment was conducted at the apple orchard base in Zhudian Town, Zhuanglang County, Pingliang City, Gansu Province (105°07′ E, 35°22′ N). The site features a temperate continental monsoon climate with an elevation of 1963 m. The field experiment began in late March 2023 and continued through October 2025. The year 2023 was the establishment year, and 2024 and 2025 were the two observation years. Soil and fruit samples used in this study were collected in 2024 and 2025.

The climatic conditions during the study period were as follows: in 2023, the average annual temperature was 9.22 °C, with 2745.45 h of sunshine and 455.6 mm of precipitation; in 2024, the average annual temperature was 9.20 °C, with 2090.6 h of sunshine and 420.6 mm of precipitation; in 2025, the average annual temperature was 9.80 °C, with 1879.3 h of sunshine and 540.5 mm of precipitation. The soil is primarily loess, characteristic of the terraced fields found in the hilly and gully-ridden regions of the Loess Plateau. It ranges in texture from clay loam to sandy loam, has a loose structure, and exhibits moderate water and nutrient retention, though its organic matter content is relatively low.

### 4.2. Experimental Materials and Treatments

Six-year-old ‘Yanfu 3’ grafted seedlings on M9-T337 rootstock, planted in a north–south row orientation with a spacing of 2 m × 4 m and trained in a spindle shape. Annual ryegrass (*Lolium perenne* L.), Perennial ryegrass, White clover (*Trifolium repens* L.), Smooth vetch (*Vicia villosa* Var.), Common vetch (*Vicia sativa* L.), Rapeseed (*Brassica napus* L.), Hairy vetch (*Vicia villosa* Roth.), and Cocksfoot (*Dactylis glomeratai* L.) were selected as green manure crops. Clean tillage was used as the control (CK), and the eight treatment groups were labeled T1 through T8, respectively. All seeds were purchased from Shuyang County Lvtong Landscape Engineering Co., Ltd. (Suqian, China).

The field experiment began in late March 2023 (establishment year) After rainfall, seeds were sown in rows 1 m from the fruit tree trunks at a depth of 1.5–3 cm, with sowing rates of 30 kg ha^−1^ for annual ryegrass, perennial ryegrass, and cocksfoot; 37.5 kg ha^−1^ for white clover and rapeseed; and 75 kg ha^−1^ for smooth vetch, common vetch, and hairy vetch. After sowing, the seeds were covered with 1–2 cm of fine soil, which was lightly compacted to ensure close contact between the soil and the seeds. Each treatment had a plot area of 100 m^2^ (50 m × 2 m), with three replicates. A row of fruit tree isolation belts was set up between plots. The coverage rate of each grass species exceeded 80%, and the grass strip was maintained 1 m away from the tree base, resulting in a grass width of approximately 2 m. After grass emergence, quick-acting fertilizer (urea, 75–120 kg/ha) was applied once or twice to promote grass growth. When the grass reached a height of 30–40 cm, it was cut in time, leaving a stubble height of 10 cm. At that time, quick-acting fertilizer was applied once. In August 2023 and 2024, residues were incorporated by rotary tillage. Soil and fruit samples used in this manuscript were collected in 2024 and 2025. Throughout the entire growth period of the grass species, weeds were promptly removed to prevent any from growing. The remaining orchard management measures followed the routine practices of the fruit growers. A linear mixed-effects model was used with treatment, year, and treatment × year as fixed effects and block as random effects, Tukey’s HSD was used for multiple comparisons.

### 4.3. Soil Sampling Analysis

Five trees were selected from each treatment area. Soil samples were collected once a month during the growing season (from April to October) in each sampling year. However, only the October samples were used for the analyses presented in this study, because October represents the end of the growing season and the period most closely associated with fruit maturation and quality. Soil samples were collected from the 0–20 cm depth layer within a 50 cm radius from the root of each apple tree using the five-point sampling method. Three replicates were established, and five soil samples were taken from each replicate and subsequently mixed. The mixed soil samples were divided into two portions. One portion was used to analyze physicochemical properties, including pH, soil organic matter, total nitrogen, total phosphorus, and total potassium. The other portion was stored in 2 mL centrifuge tubes and transported on dry ice for the determination of soil microbial community structure.

### 4.4. Soil Microbial Diversity Analysis

DNA was extracted from 0.5 g soil using the E.Z.N.A.^®^ Soil DNA Kit (Omega Bio-tek, Norcross, GA, USA). For bacterial communities, the V3–V4 region of the 16S rRNA gene was amplified with primers 338F (5′-ACTCCTACGGGAGGCAGCA-3′) and 806R (5′-GGACTACHVGGGTWTCTAAT-3′). For fungal communities, the ITS1 region was amplified with primers ITS1F (5′-CTTGGTCATTTAGAGGAAGTAA-3′) and ITS2 (5′-GCTGCGTTCTTCATCGATGC-3′). PCR reactions containing 4 μL 5× FastPfu buffer, 2 μL dNTPs (2.5 mM), 0.8 μL each primer (5 μM), 0.4 μL FastPfu polymerase, and 10 ng template, under 95 °C 3 min; 27 cycles of 95 °C 30s, 55 °C 30s, 72 °C 45s; and 72 °C 10 min. Amplicons were pooled equally and sequenced (2 × 300 bp) on an Illumina MiSeq platform (Biomarker Technologies, Beijing, China). Raw reads were trimmed (Trimmomatic v0.36), merged (FLASH v1.2.11; overlap ≥ 10 bp, mismatch ≤ 0.2), clustered into OTUs at 97% similarity (UPARSE v7.1), and checked for chimeras (UCHIME v4.2). OTU taxonomy was assigned using RDP Classifier v2.13 against SILVA v138. Alpha diversity (Chao1, Shannon, Simpson) and beta diversity (Bray–Curtis PCoA) were calculated with Mothur v1.30.2 and R/vegan, respectively.” Functional predictions were performed using FAPROTAX (v1.2.4) for bacterial taxa and Tax4Fun (v1.0) for bacterial metabolic pathways, and FUNGuild (v1.1) for fungal trophic modes. These tools infer potential functions from taxonomic composition and do not directly measure gene expression or enzyme activity.

### 4.5. Soil Physical and Chemical Properties Analysis

In this study, total nutrient pools were measured rather than total nutrient fractions. For total elements, soil was digested with HNO_3_–HClO_4_–HF. Total N was analyzed by Kjeldahl digestion, distillation, and titration; total P by vanadomolybdate colorimetry after H_2_SO_4_–HClO_4_ digestion; and total K by ICP-OES following ammonium acetate extraction (pH 7.0). All analyses were run in triplicate, with results expressed on an oven-dry basis (105 °C, 24 h), and quality was ensured using certified reference materials and reagent blanks. The above chemicals were purchased from Shanghai Sangong Biotechnology Co., Ltd (Shanghai, China).

### 4.6. Fruit Determination Indicators and Methods

At the full fruit ripening stage, uniform and disease-free apple fruits without mechanical damage were randomly collected from four canopy orientations (east, south, west, north) of five representative trees per treatment. Thirty fruits were mixed as one composite sample for each fertilization group. All fruit samples were transported under low-light and low-temperature conditions immediately after harvest. A portion of fresh intact fruits was reserved for external quality analysis. In contrast, the remaining fruits were peeled, deseeded, and homogenized into pulp for subsequent determination of internal nutritional and flavor indicators. Three biological replicates were set for each treatment, and all measurements were performed with at least three technical replicates.

Fruit peel color (L, a, b*) was recorded on the sun-exposed surface using a CS410 colorimeter (8 mm aperture, 13 mm illumination), calibrated with black/white standards. Thirty uniform fruits were randomly picked to determine individual weight (0.01-g balance), longitudinal/transverse diameters (vernier caliper), and shape index (length/diameter ratio). Firmness was averaged from four equidistant measurements per fruit after slight peeling, using a GY-4J penetrometer. Soluble solids were measured with a PAL-1 refractometer on extracted juice, with readings taken after stabilization; each batch included six biological replicates. For biochemical components, soluble sugars (anthrone method), soluble protein (Coomassie brilliant blue G-250), titratable acidity (neutralization titration), total phenolics (Folin–Ciocalteu), vitamin C (xylene extraction colorimetry), anthocyanins and flavonoids (spectrophotometry) were quantified. Marketable yield per tree or per hectare, fruit grading, and marketable fruit rate were not determined in this study; therefore, yield-related conclusions are not drawn.

### 4.7. Statistical Analysis

Data processing was conducted using Excel 2021; chart plotting was performed with Origin 2022; significance analysis was carried out using SPSS 20.0; one-way ANOVA (analysis of variance) was employed for both variance analysis and multiple comparisons, with a significance level set at *p* < 0.05.

## 5. Conclusions

This research shows that using appropriate green manures as cover crops between rows effectively reduces soil degradation and enhances apple fruit quality on the Loess Plateau. Of the eight species evaluated, hairy vetch (T7) and white clover (T3) showed the best overall results. Hairy vetch led to the greatest increases in soil organic matter (34.1%) and total nitrogen (40.2%), while white clover significantly boosted microbial diversity and promoted beneficial genera such as Sphingomonas, Lysobacter, and Mortierella. Functional predictions based on amplicon data suggested potential shifts in microbial functions, but these require further experimental validation. Common vetch (T5) and cocksfoot (T8) were most effective in improving the sugar-acid balance of the fruit. However, because marketable yield was not measured, these results should be interpreted as improvements in fruit compositional quality rather than confirmed yield gains. Results analysis identified hairy vetch and white clover as the top choices for sustainable orchard management. Implementing these cover crops can decrease fertilizer use, reduce soil degradation, and improve both the ecological and economic sustainability of apple farming in rain-fed semi-arid areas. Future research should focus on long-term studies and mixed-species plantings to further optimize results.

## Figures and Tables

**Table 1:** Soil properties under different grass types.

Treatments	pH	SOM (g/kg)	TN (g/kg)	TP (g/kg)	TK (g/kg)
CK	8.17 ± 0.01 ^a^	19.29 ± 0.40 ^d^	1.02 ± 0.09 ^d^	0.72 ± 0.04 ^c^	11.61 ± 1.12 ^c^
T1	8.10 ± 0.02 ^bc^	21.88 ± 0.34 ^bc^	1.16 ± 0.02 ^bc^	0.97 ± 0.01 ^a^	17.35 ± 3.03 ^ab^
T2	7.99 ± 0.01 ^cd^	22.70 ± 0.30 ^b^	1.24 ± 0.08 ^bc^	0.71 ± 0.05 ^cd^	20.85 ± 0.70 ^a^
T3	8.04 ± 0.01 ^c^	25.33 ± 0.28 ^a^	1.08 ± 0.19 ^cd^	0.74 ± 0.04 ^bc^	14.78 ± 0.20 ^bc^
T4	8.11 ± 0.01 ^bc^	17.02 ± 0.58 ^e^	1.10 ± 0.04 ^bc^	0.81 ± 0.01 ^bc^	18.07 ± 0.46 ^ab^
T5	8.09 ± 0.01 ^bc^	22.25 ± 0.55 ^b^	1.13 ± 0.15 ^b^	0.84 ± 0.05 ^b^	18.53 ± 0.53 ^ab^
T6	7.82 ± 0.01 ^d^	20.66 ± 0.55 ^c^	1.16 ± 0.02 ^bc^	0.62 ± 0.05 ^d^	13.37 ± 0.41 ^bc^
T7	8.00 ± 0.01 ^c^	25.86 ± 0.60 ^a^	1.43 ± 0.01 ^a^	0.83 ± 0.02 ^b^	17.77 ± 0.05 ^ab^
T8	8.12 ± 0.02 ^b^	22.69 ± 0.23 ^b^	1.21 ± 0.01 ^bc^	0.81 ± 0.04 ^bc^	16.70 ± 0.77 ^ab^

Note: Values in the table are means ± SD (*n* = 3). CK, clean tillage; T1, annual ryegrass; T2, perennial ryegrass; T3, white clover; T4, smooth vetch; T5, common vetch; T6, rapeseed; T7, hairy vetch; T8, cocksfoot. Different lowercase letters indicate significant differences among the various green manure treatments based on the LSD test (*p* < 0.05). Overall treatment effects were tested by one-way ANOVA.

**Table 2:** Analysis of soil microbial Alpha diversity under different grass types.

	Bacterial		Fungal	
Treatments	Shannon	Chao1	Shannon	Chao1
CK	9.40 ± 0.07 ^ab^	1216.89 ± 16.97 ^de^	5.63 ± 0.96 ^bc^	348.00 ± 80.54 ^b^
T1	9.66 ± 0.27 ^ab^	1214.88 ± 22.29 ^de^	5.38 ± 0.26 ^bc^	231.00 ± 69.02 ^c^
T2	9.28 ± 0.06 ^b^	1159.46 ± 12.88 ^e^	4.77 ± 0.10 ^c^	213.00 ± 14.18 ^c^
T3	9.73 ± 0.39 ^a^	1431.08 ± 98.99 ^a^	6.78 ± 0.61 ^a^	449.00 ± 31.75 ^a^
T4	9.66 ± 0.18 ^ab^	1254.80 ± 55.40 ^cd^	4.85 ± 0.99 ^bc^	283.00 ± 28.62 ^bc^
T5	9.69 ± 0.13 ^ab^	1365.20 ± 31.33 ^ab^	5.60 ± 0.59 ^bc^	226.67 ± 33.47 ^c^
T6	9.55 ± 0.35 ^ab^	1332.83 ± 30.36 ^bc^	6.03 ± 0.51 ^ab^	352.84 ± 28.16 ^b^
T7	9.45 ± 0.10 ^ab^	1226.86 ± 51.48 ^de^	5.87 ± 0.10 ^abc^	347.00 ± 17.78 ^b^
T8	9.65 ± 0.05 ^ab^	1215.58 ± 31.83 ^de^	5.46 ± 0.60 ^bc^	276.33 ± 15.53 ^bc^

Note: Values in the table are means ± SD (*n* = 3). Different lowercase letters indicate significant differences among the various green manure treatments based on the LSD test (*p* < 0.05).

## Data Availability

The data that support the findings of this study are available from the corresponding author upon reasonable request.

## Supplementary Materials

The following supporting information can be downloaded at: https://www.mdpi.com/article/10.3390/plants15193073/s1, Figure S1: Soil microbial PCoA analysis under different grass types. Table S1: The number of soil microbial OTUs under different grass species. Table S2: Species abundance distribution at the genus level under different grass types. Table S3: Alpha Diversity of the Microbial Communities. Table S4: Soil microbial PCoA analysis under different grass types. Table S5: A predictive list of soil microbial ecological functions under different grass species.
